# Advancements in Fermented Beverage Safety: Isolation and Application of *Clavispora lusitaniae* Cl-p for Ethyl Carbamate Degradation and Enhanced Flavor Profile

**DOI:** 10.3390/microorganisms12050882

**Published:** 2024-04-28

**Authors:** Yingchun Zhao, Jun Liu, Han Wang, Fayuan Gou, Yiwei He, Lijuan Yang

**Affiliations:** 1 College of Bioengineering, Sichuan University of Science & Engineering, Yibin 644000, China; 321086002401@stu.suse.edu.cn (Y.Z.); liujunbio@suse.edu.cn (J.L.); 323095102323@stu.suse.edu.cn (H.W.); 21041040101@stu.suse.edu.cn (F.G.); 21041040102@stu.suse.edu.cn (Y.H.); 2 Liquor Making Bio-Technology & Application of Key Laboratory of Sichuan Province, Sichuan University of Science & Engineering, Yibin 644000, China

**Keywords:** ethyl carbamate, degradation *Clavispora lusitaniae*, ester production, rice wine

## Abstract

Ethyl carbamate (EC) is a natural by-product in the production of fermented food and alcoholic beverages and is carcinogenic and genotoxic, posing a significant food safety concern. In this study, *Clavispora lusitaniae* Cl-p with a strong EC degradation ability was isolated from Daqu rich in microorganisms by using EC as the sole nitrogen source. When 2.5 g/L of EC was added to the fermentation medium, the strain decomposed 47.69% of ethyl carbamate after five days of fermentation. It was unexpectedly found that the strain had the ability to produce aroma and ester, and the esterification power reached 30.78 mg/(g·100 h). When the strain was added to rice wine fermentation, compared with the control group, the EC content decreased by 41.82%, and flavor substances such as ethyl acetate and β-phenylethanol were added. The EC degradation rate of the immobilized crude enzyme in the finished yellow rice wine reached 31.01%, and the flavor substances of yellow rice wine were not affected. The strain is expected to be used in the fermented food industry to reduce EC residue and improve the safety of fermented food.

## 1. Introduction

Ethyl carbamate, also known as urethane, is a natural by-product in the production of fermented foods [1] and fermented beverages [2]. In 1943, Nettleship et al. discovered the potential carcinogenicity of EC, which is a food-borne carcinogen that can cause lymphoma, lung tumors, liver cancer, skin cancer and other diseases. In 2007, the International Agency for Research on Cancer (IARC) classified EC as Class 2A [3]. Therefore, it is of great significance to study the content control of ethyl carbamate in fermented beverage wine.

EC is formed from precursors (urea, cyanate radical, carbamoyl phosphate, citrulline and diethyl pyrocarbonate) through different processes. Once EC is formed, it is very stable and difficult to eliminate. Biological enzymatic methods (acid urease and urethanase) have the characteristics of high efficiency, strong specificity and mild action conditions, which make it a focus in solving the problem of EC in alcoholic beverages. Therefore, in 1990, Kobashi [4], a Japanese scholar, identified *Citrobacter* sp. in the mouse intestine. After the first discovery and confirmation of urethanase that can degrade EC, a large number of scholars have conducted a lot of research on EC degradation. So far, however, many strains that degrade EC have been found, including *Bacillus licheniformis* 1013 and *Bacillus licheniformis* 12107 [5,6], *Marine micrococcus* [7] and *Rhodococcus equistrain* TB-60 [8], *Rhodotorula* sp. [9], *Penicillium variabile* [10], *Klebsiella pneumoniae* [11], *Lysinibacillus fusiformis* SC02 [12], *Exiguobacterium* sp. Alg-S5 [13], *Meyerozyma caribbica* SKa5 [14], *Candida parapsilosis* [15], *Aspergillus oryzae* [16], *Agrobacterium tumefaciens* d3 [17], and *Enterobacter* sp. R-SYB082 [18]. However, only *Rhodococcus equistrain* TB-60, *Lysinibacillus fusiformis* SC02 *Acinetobacter calcoaceticus*, *Candida parapsilosis*, *Aspergillus oryzae*, *Agrobacterium tumefaciens* d3 and *Enterobacter* sp. R-SYB082 obtained the gene sequence of urethanase. However, even though urethanase can be expressed heterologously, the problems of urethanase, such as low affinity for EC, acid resistance and ethanol resistance, cannot be applied to degrade trace EC in alcoholic beverages (rice wine, yellow rice wine, Baijiu, etc.), and the problem of EC in fermented beverage wine has not been well solved. In view of this problem, in order to improve the safety and quality of fermented wine (wine, rice wine, yellow rice wine and Baijiu) in China, in recent years, people began to consider breeding fermentation strains with low urea and ethyl carbamate to be used in the production and fermentation process. Among the many above-mentioned strains that degrade ethyl carbamate, except *Rhodococcus equi*, *Klebsiella pneumoniae*, *Acinetobacter calcoaceticus* and *Candida parapsilosis*, most of them are bacteria, and the fermentation ability of bacteria in wine brewing is far less than mold and yeast, and wine brewing only needs yeast. Therefore, it is necessary to screen out more beneficial yeast with EC degradation ability and higher application value in the production of wine, rice wine, yellow rice wine and Baijiu.

Presently, only two strains of yeast, *Meyerozyma caribbica* SKa5 and *Candida parapsilosis*, have been identified, with *Candida parapsilosis* being a conditional pathogen [19] that may not be entirely safe for actual production processes. Therefore, this study screened *Clavispora lusitaniae* Cl-p for its strong EC degrading ability. Some studies have shown that *Clavispora lusitaniae* is an ester-producing yeast; Lin [20] used it as a wine starter to ferment fruit wine, and Jiang [21] combined it with other strains to prepare a microecological preparation of traditional Chinese medicine for preventing and treating diarrhea in ruminants. Consequently, the application of *Clavispora lusitaniae* in the brewing process of fermented wine can help control EC content, enhance the flavor profile of the wine body and improve overall safety. We aim to make practical contributions to reducing the EC level in yellow rice wine, rice wine, Baijiu, wine and other fermented wine, so as to promote the healthy development of the fermented food industry in China.

## 2. Materials and Methods

### 2.1. Materials and Reagents

Microorganisms come from Daqu in a winery in Yibin. Daqu is crushed into powder and packed in clean fresh-keeping bags. The ethyl carbamate is from Macklin Co., Ltd., Shanghai, China and the rice leaven Angel Yeast Co., Ltd., Yichang, China.

### 2.2. Culture Medium

YPD medium (Sangon Co., Ltd., Shanghai, China); the YPD solid medium needs 25 g/L agar powder (AOBOX Co., Ltd., Beijing, China).

Nitrogen source screening medium (g/L): 2 glucose (AOBOX Co., Ltd., Beijing, China), 5 ethyl carbamate (Macklin Co., Ltd., Shanghai, China), 2 sodium acetate (Chron Chemicals Co., Ltd., Chengdu, China), 5 NaCl (Chron Chemicals Co., Ltd., Chengdu, China), 2 KH_2_PO_4_ (Chron Chemicals Co., Ltd., Chengdu, China), 0.005 Bromocresol Violet (Chron Chemicals Co., Ltd., Chengdu, China), pH natural.

Fermentation medium (g/L): 2 glucose (AOBOX Co., Ltd., Beijing, China), 2.5 ethyl carbamate (Macklin Co., Ltd., Shanghai, China), 2 NaCl (Chron Chemicals Co., Ltd., Chengdu, China), 1 peptone (AOBOX Co., Ltd., Beijing, China), 2.5 KH_2_PO_4_ (Chron Chemicals Co., Ltd., Chengdu, China), pH natural.

Wort medium (g/L): 50 malt powder (Solarbio Co., Ltd., Beijing, China), 10 yeast powder (AOBOX Co., Ltd., Beijing, China), pH natural.

Enzyme producing medium (g/L): 250 bran (Yibin, China), distilled water 0.1, pH natural.

The above mediums were sterilized at 115 °C for 30 min.

### 2.3. Screening of Ethyl Carbamate-Degrading Strains

Weigh 10 g Daqu powder and put it in 90 mL sterile normal saline (250 mL triangular bottle), shake it for 2 h at 30 °C and 180 r/min, absorb 1 mL enriched bacterial solution and add it to 9 mL of normal saline, to obtain different dilution gradients (10^−1^, 10^−2^, 10^−3^, 10^−4^, 10^−5^, 10^−6^, 10^−7^). Select bacterial liquid with dilutions of 10^−4^, 10^−5^, 10^−6^ and 10^−7^ and coat it on YPD solid medium for streaking separation (purification for 2–3 times) until a typical single colony is selected.

Initial screening: The single colonies isolated were selected, inoculated with YPD liquid medium for activation, then inoculated with 2% (*v*/*v*) in nitrogen source screening medium, oscillated for 2 d at 30 °C, 180 r/min, and screened by color reaction (the dark ones were selected).

Re-screening: The initially screened colonies were inoculated on fermentation medium (control group: fermentation medium without adding strains), oscillated at 30 °C, 180 r/min for 5 d, and centrifuged at 7000 rpm for 10 min. The sample was pretreated by the improved method [22] and passed through a 0.22 μm filter membrane. GC-MS [22]: DB-WAX column (60 m × 0.25 mm × 0.25 μm) and the inlet temperature was 250 °C. The initial temperature was kept at 50 °C for 1 min, then increased to 180 °C at 8 °C/min for 20 min and then kept at 240 °C for 5 min. The solvent delay was 17 min. Carrier gas: high purity He, flow rate 1.5 mL/min and shunt injection 10:1. The sample size was 1 μL. Mass spectrum conditions: electron energy: 70 eV; transmission line temperature: 250 °C; ion source temperature: 230 °C; quadrupole temperature: 150 °C. Detection methods: select ion detection (SIM), ethyl carbamate select monitoring ion (*m*/*z*): 62.0, 74.0, 89.0, quantitative ion 62.0; and D_5_-ethyl carbamate-monitoring ions (*m*/*z*): 64, 76, quantitative ions 64.0. The content of EC in the fermentation medium was detected, and the strains with a strong ability to degrade EC were screened out.

### 2.4. Identification of Strains

Morphological identification: The isolated strains were marked on YPD solid medium, and the colony morphology was observed with the naked eye. Single colonies were selected for Meilan staining, and their morphological characteristics were observed using an optical microscope.

Molecular biological identification: the DNA of the strain was extracted by Solarbio’s fungal genomic DNA extraction kit and amplified by PCR using universal primers ITS1 (5′-CTTGGTCATTTAGAGAGGAAGTAA-3′) and ITS4 (5′-TCCTCCTCCTCCGGTTGATTATGC-3′). PCR amplification procedure: pre-denaturing at 94 °C for 3 min, denaturing at 94 °C for 30 s, and annealing at 55 °C for 30 s and 72 °C for 1 min. After 30 cycles, it was extended to 72 °C for 10 min and finally stored at 4 °C. The PCR products were sequenced, and the sequencing results were compared and analyzed by blast provided by National Center for Biotechnology Information (NCBI), and then the phylogenetic tree was constructed by the Neighbor-Joining method using MEGA7.0 software to judge its species.

### 2.5. Analysis on the Tolerance and Fermentation Performance of the Strain

After activating the strain in YPD liquid medium, the cell concentration reaches 4.47 × 10^8^ CFU/mL, inoculated with a 5% (*v*/*v*) dosage in YPD medium with different ethanol content (0%, 5%, 7%, 8%, 9%, 10%), different NaCl content (0%, 5%, 10%, 12%, 13%, 14%, 15%) and different pH (3, 4, 5, 6, 7, 8, 9) at 30 °C. The biomass of OD_600_ was measured by oscillating culture at 180 r/min for 24 h to explore the tolerance of the strain. (YPD medium with different contents of ethanol, NaCl and pH without added yeast was used as the blank control).

Referring to the method of Zou Mouyong [23], the activated strain (cell concentration is the same as Section 2.5) was inoculated into wort medium with an inoculation amount of 2% (*v*/*v*) for culture, fermented at 30 °C and 150 r/min for 4 d, and then the aroma was evaluated. Then, the activated strain (cell concentration is the same as Section 2.5) was inoculated in the enzyme-producing culture medium with an inoculation amount of 5% (*v*/*v*), incubated at 35 °C for 7 d, then exposed to the sun to dry and stored for later use. The liquefaction enzyme, saccharification enzyme and esterification enzyme were analyzed by QB/T 4257-2011 [24].

### 2.6. Application of Cl-p Strain in Rice Wine Fermentation

The method of Gui Jiangping [25] was used to simulate rice wine brewing (the control group was set respectively: adding 4 g rice leaven to 1 kg glutinous rice; experimental groups: add 8 mL of the Cl-p strain with the same cell concentration as 2.5 to 1 kg glutinous rice and rice leaven with the same as that of the control group). After fermentation, the fermentation mash of the above groups was filtered, and the prepared filtrate (rice wine) was stored at 4 °C. The filtered rice wine was centrifuged at 7000 rpm for 10 min. The urea content is determined according to the diacetyl oxime method in GB/T18204.2-2014 [26]. The improved Mo et al. [27] method was used to pretreat rice wine samples with a 0.22 μm filter membrane. The content of ethyl carbamate and its flavor composition in the prepared rice wine were respectively detected by GC-MS (same method as Section 2.3) [28].

### 2.7. Application of Cl-p Strain Crude Enzyme in Finished Yellow Rice Wine

Preparation and immobilization of crude enzyme: The 20 mL (cell concentration is the same as Section 2.5) bacterial suspension (1 g/mL) was crushed by ultrasound to obtain the crude enzyme, which was then immobilized by the glutaraldehyde/calcium alginate capsule method [29] and fully mixed with 20 mL sodium alginate solution (40 g/L). Then, a syringe was used to add calcium chloride (20 g/L) solution at a rate of 4–8 drops/s, calcified overnight, filtered to obtain immobilized pellets, washed with deionized water, placed in 0.2% glutaraldehyde solution and shaken for 2 h at 30 °C. The control group included the inactivated 20 mL (cell concentration of the same as Section 2.5) bacterial suspension (1 g/mL) and the crude enzyme solution obtained by ultrasonic crushing, and 20 mL ultra-pure water was fully mixed with 20 mL sodium alginate solution (40 g/L), respectively, and the immobilized pellets were prepared by the above operation. The immobilized crude enzyme solution, immobilized inactivated crude enzyme solution and immobilized ultra-pure water were obtained by washing the sediment repeatedly with deionized water.

Refer to the method of Liu Qingtao [30] and make a slight modification: The strain crude enzyme and immobilized strain crude enzyme were added to yellow rice wine samples (artificially added to an EC content of 500 µg/L, pH 4.5) with a final concentration of 5000 U/L and a corresponding amount of immobilized enzyme solution, respectively, and oscillated at 37 °C for 48 h. The change in the EC content in samples and the effect of the addition of the strain crude enzyme on the flavor substances of yellow rice wine were detected. (The control group was immobilized pellets prepared by adding the inactivated strain crude enzyme and ultra-pure water; same detection method as Section 2.6).

## 3. Results and Discussion

### 3.1. Isolation and Screening of Ethyl Carbamate-Degrading Strains

**Figure 1:**
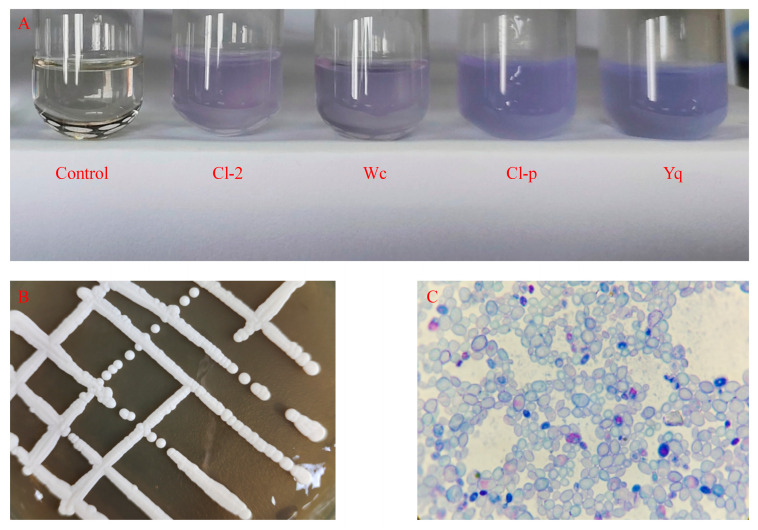
Screening, morphological characteristics and identification of ethyl carbamate-degrading strains; (**A**) color changes of different strains using medium (EC as nitrogen source); (**B**) morphological characteristics of Cl-p colonies observed with naked eye; (**C**) microscopic observation of characteristics of Cl-p strains stained by Meilan (16 × 40).

The strains for preliminary screening were selected based on the color change in bromocresol violet from yellow to purple, which is caused by the reaction between amidease or urethanase and EC resulting in ammonia production [31]. In this experiment, 14 strains of yeast were isolated and purified from Daqu. Four strains were selected from the initial screening with darker color changes in the culture medium. These strains, namely Cl-2, Wc, Cl-p and Yq, are shown in Figure 1A.

The potential target strains obtained from the preliminary screening were inoculated into the fermentation medium for 5 d, and then the consumption of ethyl carbamate in the fermentation liquid was detected by GC-MS. As shown in Table 1, the Cl-p strain exhibited the highest degradation ability, with a degradation rate of 47.69%. The Cl-2 strain showed the lowest degradation rate at only 8.4%, while the Wc and Yq strains had degradation rates of 15.6% and 20.8%, respectively. Because the Cl-p strain has the best degradation ability of ethyl carbamate, it is taken as the follow-up research object.

### 3.2. Identification of Cl-p Strain

**Figure 2:**
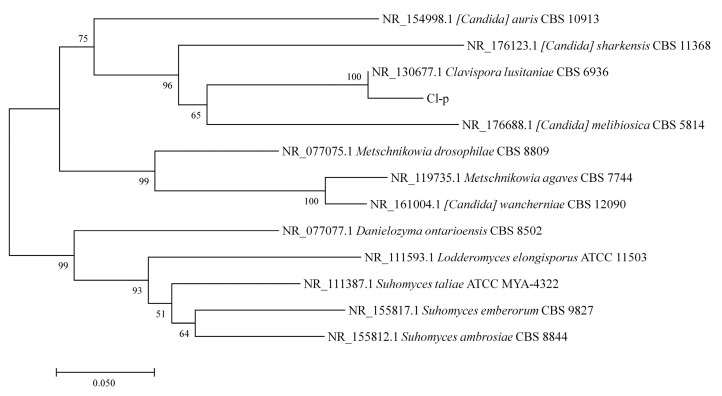
Phylogenetic tree of Cl-p strain.

The Cl-p strain was inoculated on YPD solid culture medium to examine its appearance and morphology, as illustrated in Figure 1B; the strain exhibited vigorous growth, forming a swollen, thick, milky white colony with a distinctly circular edge. The microscopic observation of the Cl-p strain after staining with methylene blue (Figure 1C) revealed a blue oval sphere. The DNA of the Cl-p strain was extracted, followed by the amplification of its ITS sequence, and the species of the Cl-p strain was inferred by Blastn sequence comparison analysis. The results showed that the ITS sequence of the Cl-p strain had the highest homology with 98.45% similarity between the Cl-p strain and *Clavispora lusitaniae.* We attempted to submit the ITS sequencing results of this strain to NCBI and obtained the login number PB2733. As shown in Figure 2, the Cl-p strain and *Clavispora lusitaniae* were clustered in the same branch and were closely related, so they were identified as *Clavispora lusitaniae*. The Cl-p strain was stored in the Strain Preservation Center of Wuhan, China with the storage number CCTCC M 20232114.

### 3.3. Analysis of Tolerance and Fermentation Performance of Cl-p Strain

**Figure 3:**
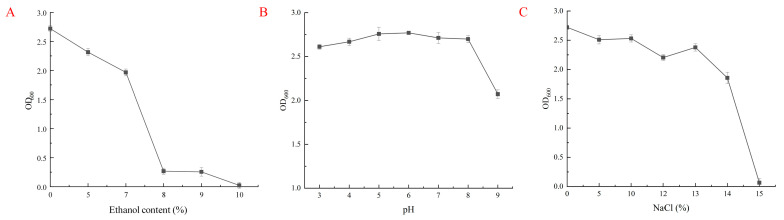
Tolerance of Cl-p strain: (**A**) ethanol resistance; (**B**) pH resistance; and (**C**) salt tolerance.

The tolerance of the Cl-p strain to ethanol, NaCl and pH was investigated; the experimental data were plotted in the form of “mean ± SD”. It can be seen from Figure 3A that the Cl-p strain can grow normally within a pH range of 3.0 to 9.0, the optimal growth concentration is at pH 6.0 and the strain can still grow normally at pH 3.0. As can be seen from Figure 3B, with the increase in alcohol concentration, the bacterial concentration of the Cl-p strain showed an overall trend of decline, and the strain could grow normally under the conditions of 0~7% ethanol concentration, while the strain could still grow when the ethanol concentration was 9%. As can be seen from Figure 3C, the Cl-p strain can grow normally when the NaCl content is 0% to 14% but almost stops growing when the NaCl content is 15%; it shows that the Cl-p strain has a strong stress resistance, can survive in high acidity, high ethanol concentration and a high permeability environment, and has the potential to be used in fermented food.

Next, the fermentation performance of the Cl-p strain was investigated by making bran Qu. The experimental data are expressed as “mean ± SD”. As shown in Table 2, the liquefaction power of the Cl-p strain was 0.45 mg/(g·h), and the saccharification power was 271 mg/(g·h). Compared with the fermentation properties measured by Yang Yazhen [32] (a liquefaction power of 0.89 mg/(g·h) and saccharification power of 267 mg/(g·h)), the liquefaction power was lower and the saccharification power was slightly higher. Zhang Zhigang [33] determined that the liquefaction power of traditional Daqu was only 0.66 mg/(g·h). The esterification power of the Cl-p strain was 30.78 mg/(g·100 h), which was in line with the 30 mg/(g·100 h) stipulated in Article 244 of the enterprise standard QB/T5188-2017 Brewing Red Yeast [34]. We expect that the next experiments with *Clavispora lusitaniae* will improve the flavor and quality of rice wine [35].

### 3.4. The Utilization of the Cl-p Strain in the Fermentation Process of Rice Wine

#### 3.4.1. Contribution of Cl-p Strain to Decrease in EC Content in Rice Wine Fermentation

**Figure 4:**
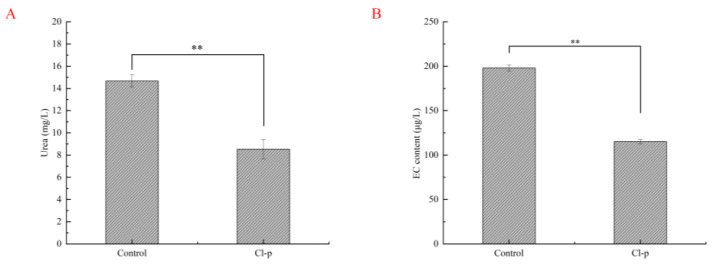
Changes in urea and EC content in rice wine prepared by adding Cl-p strain, ** *p* ≤ 0.01. (**A**) Changes in urea content; (**B**) Changes in EC content.

The effect of adding *Clavispora lusitaniae* Cl-p on the urea and EC content in simulated rice wine fermentation is shown in Figure 4. Urea is one of the precursor substances of EC, and studies have shown that the reaction between urea and ethanol is the main pathway for producing EC in fermented wine. It was found that the urea content in the experimental group was 8.54 mg/L (Figure 4A), which was 42.14% lower than the urea content in the control group (14.67 mg/L). It was found that the generation of EC in the control group was 198.03 μg/L, while the content of EC in the rice wine prepared by the experimental group was significantly reduced compared to the control group, only 115.22 μg/L (Figure 4B), with a degradation rate of 41.82%. According to statistics, the EC content in yellow rice wine produced in China is the highest, about 100–750 µg/L [36], and the EC content in rice wine is about 13–575 μg/L [37]. Researchers have tested the changes in the urea and EC content during the fermentation process of yellow rice wine, indicating a certain positive correlation between urea and EC [38]. In this study, the addition of the Cl-p strain significantly reduced the content of EC after simulating rice wine fermentation, which may be due to the decomposition and utilization of some precursor substances like urea by the strain, resulting in a decrease in EC. It is also possible to decompose urea while also decomposing the formed EC. Yu [39] inoculated *Lactobacillus brevis* as an enhanced strain into the fermented rice wine Qu, which could reduce the EC content by 40%. Jia [40] produced Baiqu-fermented Redqu yellow rice wine by adding *Rhizopus oryzae* JQA3; the EC content decreased by 39.13%, but there was a negative correlation between the urea content and EC content; Yu [41] co-fermented yellow rice wine by adding *Saccharomyces cerevisiae* and Qu, and detected a decrease of 70.06% and 68.73% in urea and EC concentrations, respectively. The ability of *Clavispora lusitaniae* Cl-p to degrade EC and urea is higher than that of *Rhizopus oryzae* JQA3 and *Lactobacillus brevis* and lower than that of *Saccharomyces cerevisiae*. However, by adding *Clavispora lusitaniae* Cl-p to simulate rice wine fermentation, the content of EC in the rice wine fermentation process can be well controlled.

#### 3.4.2. Effect of Cl-p Strain on Flavor Substances of Rice Wine

The flavor composition and changes in the prepared rice wine (control group and experimental group) were detected by GC-MS, and the influence of adding *Clavispora lusitaniae* Cl-p on the flavor of rice wine was explored. As can be seen from Table 3, a total of 35 flavor substances were detected, and the main flavor substances included 7 alcohols, 4 esters, 9 acids, 8 aldehydes, 5 ketones and 1 alkene. A total of 22 species were detected in the control group and 28 in the experimental group. It included the increase in three higher alcohols (2-methyl-1-Propanol, 3-methyl-1-Butanol, 2-Phenylethyl Alcohol) and three esters (Ethyl Acetate, 2-Hydroxyacetic acid ethyl est, Methyl 6-oxoheptanoate). Unexpectedly, it was found that β-phenylethanol could be produced by adding *Clavispora lusitaniae* to simulate fermentation. β-phenylethanol is an aromatic alcohol with the fragrance of roses, which is not only an important aroma component of Daqu but also an important component and precursor of liquor flavor. Fan [35] et al. found that *Clavispora lusitaniae* has a high yield of phenylethanol and can produce a small amount of ethyl acetate without adding acetic acid, which has potential application value in the development of sweet liquor. Ming Hongmei [42] found that *Wickerhamomyces anomalus* could produce β-phenylethanol and optimized the fermentation conditions. *Wickerhamomyces anomalus* is an important aromatic yeast and has been applied to various fermented foods and fermented beverages [43,44]. *Clavispora lusitaniae* is also a flavor-producing yeast and is also confirmed in patent CN 111363686 B [20] as a flavor-producing yeast. *Clavispora lusitaniae* has a good ability to produce alcohol [45], and it can also produce some esters, aldehydes and other flavor compounds, which can give liquor, rice wine, wine and other alcoholic beverages unique characteristics [46]. The fermentation of rice wine with *Clavispora lusitaniae* Cl-p also produced ethyl acetate (floral, honey, caramel flavor), phenylacetaldehyde (fruity, wine flavor) and other important flavor substances. These compounds can affect the quality of rice wine. *Clavispora lusitaniae* Cl-p applied to rice wine production can not only reduce the content of EC in the fermentation process and improve the safety of rice wine but also has potential application value in improving the quality of rice wine.

### 3.5. Application of Cl-p Strain Crude Enzyme in Finished Rice Wine

#### 3.5.1. Degradation of EC in Finished Yellow Rice Wine by Crude Enzyme of Cl-p Strain

Earlier, we investigated the ability of the Cl-p strain to decompose EC in the rice wine fermentation system and found that it could effectively reduce the content of EC in the simulated fermentation process. We speculated that the Cl-p strain might contain urethanase. Therefore, the Cl-p strain crude enzyme and immobilized crude enzyme were added to the finished yellow rice wine, and the application effect of its enzyme solution was explored through the degradation effect test of EC in yellow rice wine, which laid the foundation for the mining of functional genes of EC degradation in the Cl-p strain and also provided experimental data for the application of urethanase.

**Figure 5:**
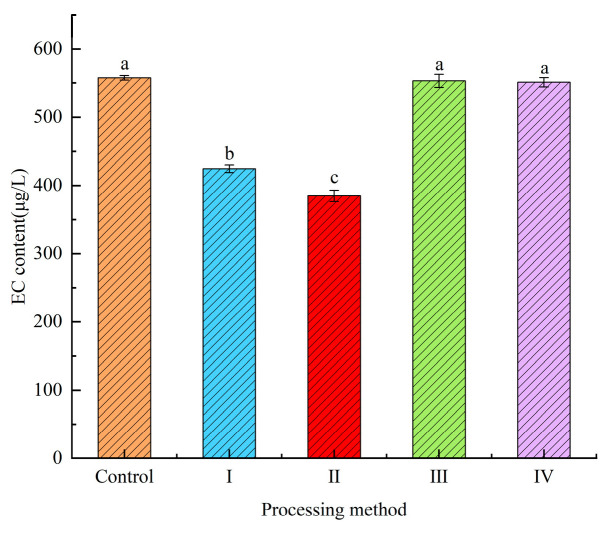
Changes in EC content in yellow rice wine treated with crude enzyme and immobilized enzyme at 37 °C for 48 h (^a,b,c^ *p* < 0.05); “Control” indicates untreated. “I” indicates crude enzyme treatment; “II” indicates immobilized crude enzyme treatment; “III” indicates inactivated crude enzyme treatment; “IV” indicates immobilized ultra-pure water treatment.

The effect of EC decomposition on finished yellow rice wine (pH 4.5, 15% ethanol concentration, *v*/*v*) treated with the Cl-p strain crude enzyme and immobilized crude enzyme for 48 h (Control is the control, I is the crude enzyme treatment, II is the immobilized crude enzyme treatment, III is the inactivated crude enzyme treatment, IV is the immobilized ultra-pure water treatment) is shown in Figure 5. The EC content of the control group was 557.52 µg/L, and the EC content of the yellow rice wine treated with III and IV was barely different from that of the control group, while the EC content of the yellow rice wine was reduced by 132.83 µg/L and 172.9 µg/L after the crude enzyme and the immobilized enzyme treatment, respectively, and the degradation rate reached 23.83% and 31.01%, respectively. Compared with the unimmobilized enzyme, the degradation effect of the immobilized enzyme is better, which may be due to the concentration of ethanol in yellow rice wine and the acidic environment limiting the ability of the unimmobilized enzyme to degrade EC, while the immobilized enzyme can improve the stability and adaptability of the enzyme to the environment and improve its ability to degrade EC. Zhou [10] added purified enzymes from *Penicillium vaginata* to wine and added 0.9 U/mL of enzymes to remove 35% of EC in wine. Dong [47] immobilized urethanase purified from Acinetobacter calc-acetoacetate after treating liquor for 12 h. The degradation rates of EC by the immobilized enzyme and unimmobilized enzyme were 65.5% and 64.8%, respectively. The ability of the immobilized and unimmobilized enzyme to degrade EC in the Cl-p strain was inferior to that of purified urethanase. It may be that the enzyme system of the crude enzyme is relatively complex, and the coordination or inhibition of multiple enzymes leads to a poor degradation effect. Therefore, the specific mechanism of Cl-p strain-mediated EC degradation needs to be further studied.

#### 3.5.2. Influence of Cl-p Strain Crude Enzyme on Flavor Substances of Finished Yellow Rice Wine

The flavor components of yellow rice wine are a complex consisting of volatile components such as alcohols, esters, ketones, phenols, organic acids, polyphenols, amino acids, sugars and other substances [48]. As an important component of yellow rice wine flavor, volatile flavor substances play a decisive role in the typicality of yellow rice wine. Through the study of volatile flavor substances in yellow rice wine, it was found that ethyl acetate, isobutanol, ethyl lactate vinegar, acetic acid, isoamyl alcohol and β-phenylethanol are the main volatile flavor substances in yellow rice wine [49,50,51], mainly alcohol esters, which affect the flavor and taste of yellow rice wine.

**Figure 6:**
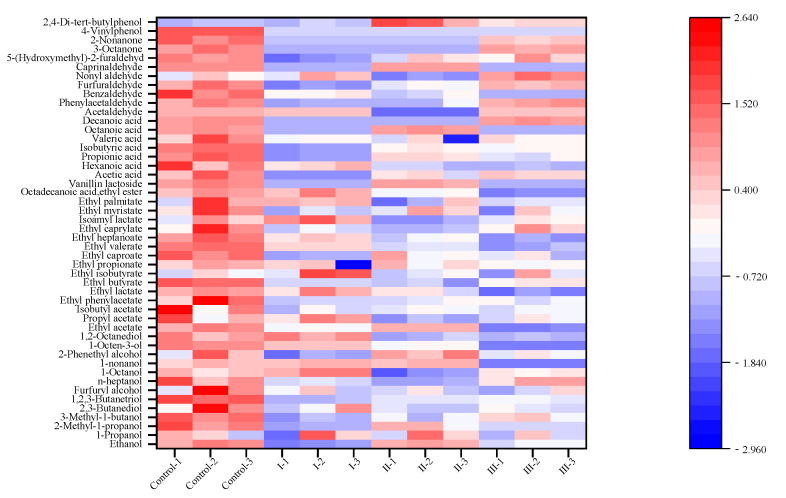
The heatmap of flavors’ substances in yellow rice wine after treatment with the crude enzyme and immobilized enzyme and at 37 °C for 48 h. The heatmap of the 13 alcohols, 17 esters, 7 organic acids, 7aldehydes, 2 ketones and 2 phenols is shown with separated colors on the left side. “Control-l, 2, 3” represents the control group. “I-1, 2, 3” indicates the crude enzyme treatment; “II-l, 2, 3” indicates the immobilized crude enzyme treatment; “III-1, 2, 3” indicates the inactivated crude enzyme treatment.

As shown in Table S1 and Figure 6, 48 flavor substances were detected in yellow rice wine by gas chromatography–mass spectrometry (GC-MS), including 13 alcohols, 17 esters, 7 acids, 7 aldehydes, 2 ketones and 2 phenols. β-phenylethanol (86.22 ± 1.02 mg/L in the control group) was the highest flavor substance in the sample, followed by isoamyl alcohol (73.78 ± 0.30 mg/L in the control group). Compared with the control group, the contents of octanol, nonyl alcohol, octanediol, isoamyl lactate and propanol in yellow rice wine treated with I (crude enzyme) increased slightly, while capric acid, 3-octanone, 2-nonone and 4-vinylphenol disappeared. The contents of propanol, β-phenylethanol, nonyl alcohol and caprylic acid in yellow rice wine treated with II (immobilized crude enzyme) were slightly increased, while 3-octanone, 2-nonone and 4-vinylphenol were not detected. The contents of propanol and nonylaldehyde in yellow rice wine treated with III (inactivated crude enzyme) were slightly increased, while the contents of nonyl alcohol, 1-octene-3-ol, caprylic acid, caprylic acid and 4-vinylphenol were not detected, and the contents of other substances were slightly decreased but within the threshold range [52].

In general, the total content of flavor substances in yellow rice wine before and after treatment with immobilized enzyme and unimmobilized enzyme had little effect on the flavor of yellow rice wine [10]. Dong [47] verified that esterase could degrade ester content in Baijiu, while the reduction in ester substances in yellow rice wine in this study was no more than 2 mg/L. Therefore, we concluded that the Cl-p strain may contain either amidase or esterase.

## 4. Conclusions

In this study, *Clavispora lusitaniae* Cl-p with a strong ability to degrade EC was isolated from Nongxiangxing Daqu using a screening medium with EC as the only nitrogen source, and the degradation rate of EC (2.5 g/L) in the fermentation medium of this strain could reach 47.69%. *Clavispora lusitaniae* Cl-p has good acid resistance, ethanol resistance and salt resistance. *Clavispora lusitaniae* Cl-p, an aroma-producing yeast, was applied to simulated rice wine fermentation. The EC content of the prepared rice wine was measured at 115.22 μg/L, which falls below the standard EC content in distilled wines from the United States and France (less than 150 ug/L). Moreover, a significant reduction of 41.82% in the EC content was observed compared to the control group. The change in the flavor substances in rice wine was detected. Due to the addition of *Clavispora lusitaniae* Cl-p, important flavor substances including ethyl acetate, β-phenylethanol, phenylacetaldehyde, isobutanol and isoamyl alcohol were increased. We hypothesized that *Clavispora lusitaniae* Cl-p would secrete urethanase and subsequently investigated the efficacy of its crude enzyme solution in degrading EC in finished yellow rice wine. The immobilized crude enzyme solution exhibited a degradation rate of 31.01% for EC without compromising the sensory attributes of yellow rice wine.

At present, we have conducted preliminary research on the application of EC-degrading strains in the brewing process and the presence of urethanases in this strain. In subsequent research, it is necessary to explore the functional genes of Cl-p strains degrading urethanases, analyze the EC degradation pathway, clarify the mechanism of *Clavispora lusitaniae* Cl-p degrading EC, promote its application in fermented food and ensure food safety.

## Figures and Tables

**Table 1:** Degradation rate of ethyl carbamate by strains.

Strain	EC Content (g/L)	Degradation Rate (%)
Control	2.50 ± 0.03	-
Cl-2	2.29 ± 0.05	8.4%
Wc	2.11 ± 0.03	15.6%
Cl-p	1.31 ± 0.04	47.69%
Yq	1.98 ± 0.02	20.8%

“-” indicates no degradation rate.

**Table 2:** Fermentation performance of Bran Qu made by Cl-p strain.

Fermenting Property	Bran Qu
liquefaction power	0.45 ± 0.01 mg/(g·h)
Saccharification power	271 ± 25.51 mg/(g·h)
esterification power	30.78 ± 0.29 mg/(g·100 h)

**Table 3:** Contents of flavors substances in rice wine prepared by adding Cl-p strain.

Compounds	Control (mg/L)	Cl-p (mg/L)
2-methyl-1-Propanol	-	0.63 ± 0.02
3-methyl-1-Butanol	-	1.95 ± 0.05
2,3-Butanediol	1.78 ± 0.03	2.21 ± 0.03
2-Furanmethanol	1.97 ± 0.05	1.77 ± 0.03
2-Phenylethyl Alcohol	-	6.88 ± 0.03
1-Propanol	1.41 ± 0.06	-
Glycerin	10.44 ± 0.10	10.25 ± 0.04
dl-Glyceraldehyde dimer	13.70 ± 0.25	-
Ethyl Acetate	-	4.80 ± 0.02
2-Hydroxyacetic acid ethyl est	-	0.11 ± 0.01
Methyl 6-oxoheptanoate	-	0.45 ± 0.02
Isosorbide Dinitrate	0.56 ± 0.04	0.50 ± 0.01
Cycloserine	0.26 ± 0.04	-
Acetic acid	5.60 ± 0.14	4.20 ± 0.01
Formic acid	1.91 ± 0.05	1.05 ± 0.02
O-(phenylmethyl)-L-Serine	0.37 ± 0.02	-
O-Acetyl-L-serine	0.77 ± 0.04	-
3-hydroxy-Dodecanoic acid	0.24 ± 0.02	0.29 ± 0.01
Muramic acid	5.54 ± 0.19	-
Alanine	-	0.06 ± 0.001
î-N-Formyl-L-lysine	-	0.22 ± 0.01
5-(hydroxymethyl)-2-Furancarboxaldehyde	2.97 ± 0.08	2.06 ± 0.02
3-(methylthio)-Propanal	-	0.16 ± 0.02
Phenylacetaldehyde	-	0.53 ± 0.02
5-methyl-2-Furancarboxaldehyde	0.50 ± 0.02	-
Furfural	0.95 ± 0.03	0.33 ± 0.02
hydroxy-Acetaldehyde	1.16 ± 0.01	0.68 ± 0.02
2-methyl-Propanal	0.89 ± 0.02	1.33 ± 0.03
Acetaldehyde	-	1.14 ± 0.03
3-hydroxy-2-Butanone	-	0.96 ± 0.02
1-hydroxy-2-Propanone	0.63 ± 0.04	0.69 ± 0.01
2-hydroxy-2-Cyclopenten-1-one	1.03 ± 0.02	0.83 ± 0.02
2,3-dihydro-3,5-dihydroxy-6-methyl-4H-Pyran-4-one	1.11 ± 0.02	0.89 ± 0.02
1,3-dihydroxy-2-Propanone	-	1.31 ± 0.02
(R*,R*)-(ñ)-2,2′-Bioxirane	1.05 ± 0.02	0.84 ± 0.02

“-” indicates no detected.

## Data Availability

Data are contained within the article and Supplementary Materials.

## Supplementary Materials

The following supporting information can be downloaded at: https://www.mdpi.com/article/10.3390/microorganisms12050882/s1, Table S1: Changes of flavor substances in finished rice wine treated by Cl-p strain crude enzyme, immobilized crude enzyme and at 37 °C for 48 h.
