# Supplementary material for: Advancements in Fermented Beverage Safety: Isolation and Application of Clavispora lusitaniae Cl-p for Ethyl Carbamate Degradation and Enhanced Flavor Profile
Source: Microorganisms. 2024 Apr 28;12(5):882. doi: 10.3390/microorganisms12050882 (PMC11124150; doi:10.3390/microorganisms12050882)
Supplement: Supplementary file 1 [file microorganisms-12-00882-s001.zip › microorganisms-2943380-supplementary.pdf]

Table S1. Changes of flavor substances in finished rice wine treated by Cl-p strain crude enzyme, immobilized crude enzyme and at 37 °C for 48 h.

Compounds	Concentration (mg/L)			
	Control	I	II	III
Ethanol	$35.40^{a} \pm 0.95$	$28.39^{c} \pm 0.64$	$34.90^{a} \pm 0.25$	$31.47^{b} \pm 0.68$
1-Propanol	$4.62^{a} \pm 0.23$	$4.74^{a} \pm 0.40$	$4.81^{a} \pm 0.25$	$4.69^{a} \pm 0.18$
2-Methyl-1-propanol	$16.75^{a} \pm 0.71$	$13.94^{b} \pm 0.13$	$15.99^{a} \pm 0.07$	$14.42^{b} \pm 0.07$
3-Methyl-1-butanol	$73.78^{a} \pm 0.30$	$71.85^{ab} \pm 0.29$	$72.14^{b} \pm 1.00$	$72.99^{ab} \pm 0.14$
2,3-Butanediol	$3.30^{a} \pm 0.19$	$3.12^{a} \pm 0.03$	$3.11^{a} \pm 0.04$	$3.15^{a} \pm 0.02$
1,2,3-Butanetriol	$2.80^{a} \pm 0.09$	$1.60^{c} \pm 0.01$	$1.85^{b} \pm 0.01$	$1.91^{b} \pm 0.05$
Furfuryl alcohol	$8.29^{a} \pm 0.35$	$8.14^{a} \pm 0.07$	$8.19^{a} \pm 0.09$	$7.98^{a} \pm 0.07$
n-heptanol	$1.17^{a} \pm 0.17$	$0.90^{a} \pm 0.01$	$1.08^{a} \pm 0.01$	$1.06^{a} \pm 0.09$
1-Octanol	$2.65^{a} \pm 0.12$	$2.81^{a} \pm 0.11$	$2.47^{b} \pm 0.13$	$2.54^{a} \pm 0.04$
1-nonanol	$1.69^{a} \pm 0.23$	$1.80^{a} \pm 0.01$	$1.80^{a} \pm 0.02$	-
2-Phenethyl alcohol	$86.22^{a} \pm 1.02$	$84.76^{a} \pm 0.38$	$86.29^{a} \pm 0.29$	$85.72^{a} \pm 0.31$
1-Octen-3-ol	$0.92^{a} \pm 0.04$	$0.70^{ab} \pm 0.01$	$0.48^{b} \pm 0.04$	-
1,2-Octanediol	$2.71^{a} \pm 0.06$	$2.73^{a} \pm 0.04$	$2.48^{b} \pm 0.03$	$2.50^{b} \pm 0.01$
Ethyl acetate	$24.43^{a} \pm 0.79$	$22.50^{b} \pm 0.18$	$23.77^{a} \pm 0.18$	$19.06^{c} \pm 0.10$
Propyl acetate	$0.27^{a} \pm 0.04$	$0.26^{a} \pm 0.02$	$0.22^{a} \pm 0.01$	$0.24^{a} \pm 0.01$
Isobutyl acetate	$0.29^{a} \pm 0.04$	$0.27^{a} \pm 0.01$	$0.27^{a} \pm 0.01$	$0.26^{a} \pm 0.01$
Ethyl phenylacetate	$5.40^{a} \pm 0.38$	$4.89^{a} \pm 0.03$	$4.93^{a} \pm 0.03$	$4.97^{a} \pm 0.09$
Ethyl lactate	$25.48^{a} \pm 0.34$	$25.27^{a} \pm 0.72$	$24.73^{ab} \pm 0.08$	$23.06^{c} \pm 0.41$
Ethyl butyrate	$27.70^{a} \pm 0.20$	$25.04^{c} \pm 0.07$	$26.82^{d} \pm 0.23$	$25.86^{b} \pm 0.13$
Ethyl isobutyrate	$1.02^{a} \pm 0.04$	$1.07^{a} \pm 0.09$	$0.99^{a} \pm 0.01$	$1.01^{a} \pm 0.10$
Ethyl propionate	$2.75^{a} \pm 0.11$	$2.70^{a} \pm 0.03$	$2.63^{a} \pm 0.18$	$2.51^{a}\pm0.03$
Ethyl caproate	$3.38^{a} \pm 0.06$	$2.90^{b} \pm 0.01$	$3.03^{b} \pm 0.07$	$3.08^{b} \pm 0.07$
Ethyl valerate	$1.58^{a} \pm 0.02$	$1.40^{b} \pm 0.01$	$1.25^{c} \pm 0.01$	$1.11^{d} \pm 0.24$
Ethyl heptanoate	$1.79^{a} \pm 0.06$	$1.68^{ab} \pm 0.03$	$1.58^{bc} \pm 0.05$	$1.48^{c} \pm 0.03$
Ethyl caprylate	$1.21^{a} \pm 0.19$	$1.01^{a} \pm 0.04$	$1.07^{a} \pm 0.02$	$1.14^{a} \pm 0.08$
Isoamyl lactate	$0.62^{ab} \pm 0.08$	$0.69^{a} \pm 0.04$	$0.50^{b} \pm 0.01$	$0.59^{ab} \pm 0.02$
Ethyl myristate	$0.50^{a} \pm 0.04$	$0.44^{a} \pm 0.02$	$0.47^{a} \pm 0.03$	$0.45^{a} \pm 0.04$
Ethyl palmitate	$1.15^{a} \pm 0.12$	$1.14^{a} \pm 0.01$	$1.01^{a} \pm 0.04$	$1.07^{a} \pm 0.01$
Octadecanoic acid,ethyl ester	$0.82^{a} \pm 0.09$	$0.82^{a} \pm 0.10$	$0.32^{b} \pm 0.01$	$0.37^{b} \pm 0.03$
Vanillin lactoside	$0.62^{a} \pm 0.10$	$0.58^{a} \pm 0.02$	$0.54^{a} \pm 0.01$	$0.34^{b} \pm 0.02$
Acetic acid	$10.37^{a} \pm 0.42$	$8.89^{b} \pm 0.03$	$9.84^{a} \pm 0.13$	$9.96^{a} \pm 0.02$
Hexanoic acid	$2.15^{a} \pm 0.18$	$1.99^{ab} \pm 0.01$	$1.65^{b} \pm 0.01$	$1.79^{ab} \pm 0.03$
Propionic acid	$0.77^{a} \pm 0.03$	$0.49^{bc} \pm 0.02$	$0.46^{c} \pm 0.01$	$0.57^{b} \pm 0.02$
Isobutyric acid	$1.01^{a} \pm 0.03$	$0.40^{d} \pm 0.02$	$0.50^{c} \pm 0.01$	$0.65^{b} \pm 0.03$
Valeric acid	$14.30^{a} \pm 0.44$	$13.84^{ab} \pm 0.09$	$13.81^{b} \pm 0.24$	$13.93^{a} \pm 0.07$
Octanoic acid	$0.78^{a} \pm 0.01$	$0.57^{a} \pm 0.02$	$0.79^{a} \pm 0.02$	-
Decanoic acid	$0.14^{a} \pm 0.02$	$0.09^{b} \pm 0.02$	$0.12^{a} \pm 0.02$	$0.13^{a} \pm 0.01$
Acetaldehyde	$5.86^{a} \pm 0.10$	$5.53^{b} \pm 0.07$	$5.51^{b} \pm 0.04$	$5.63^{ab} \pm 0.06$
Phenylacetaldehyde	$11.15^{a} \pm 0.39$	$8.87^{b} \pm 0.04$	$7.93^{c} \pm 0.06$	$10.98^{a} \pm 0.15$
Benzaldehyde	$2.87^{a} \pm 0.28$	$2.26^{b} \pm 0.03$	$2.01^{b} \pm 0.03$	$1.89^{b} \pm 0.02$
Furfuraldehyde	$0.69^{a} \pm 0.05$	$0.47^{b} \pm 0.02$	$0.57^{ab} \pm 0.03$	$0.65^{a} \pm 0.01$
Nonyl aldehyde	$0.38^{ab} \pm 0.02$	$0.37^{ab} \pm 0.03$	$0.31^{b} \pm 0.01$	$0.42^{a} \pm 0.02$
Caprinaldehyde	$0.20^{a} \pm 0.01$	-	$0.17^{a} \pm 0.01$	-
5-(Hydroxymethyl)-2-furaldehyd	$0.40^{a} \pm 0.01$	$0.30^{b} \pm 0.01$	$0.28^{b} \pm 0.01$	$0.38^{a} \pm 0.03$
3-Octanone	$0.024^{a} \pm 0.004$	-	-	$0.022^{a} \pm 0.002$
2-Nonanone	$0.015^{a} \pm 0.002$	-	-	$0.009^{b} \pm 0.001$
4-Vinylphenol	$0.020^{a} \pm 0.004$	-	-	-
2,4-Di-tert-butylphenol	$0.026^{a} \pm 0.001$	$0.025^{a} \pm 0.003$	$0.024^{a} \pm 0.002$	$0.025^{a} \pm 0.001$

Different letters in the same column indicate a significant difference (^{a,b,c,d} $p < 0.05$). Significance only indicates the comparison of the same substance in different groups. "Control" indicates untreated. "-" indicates no detected. "I" indicates crude enzyme treatment; "II" indicates immobilized crude enzyme treatment; "III" indicates inactivated crude enzyme treatment.
